# Node-RADS for preoperative locoregional nodal staging of endometrial cancer: reproducibility and accuracy assessment using CT and MRI

**DOI:** 10.1007/s00330-025-11923-4

**Published:** 2025-08-20

**Authors:** Matteo Bonatti, Riccardo Valletta, Valentina Corato, Bernardo Proner, Laurjan Hoxha, Luca Odoferdi, Martin Steinkasserer, Roberta Valerieva Ninkova, Giacomo Avesani, Vincenzo Vingiani, Lucia Manganaro

**Affiliations:** 1 Department of Radiology, Hospital of Bolzano (SABES-ASDAA), Teaching Hospital of Paracelsus Medical University (PMU), Bolzano, Italy; 2 Department of Gynecology and Obstetrics, Hospital of Bolzano (SABES-ASDAA), Teaching Hospital of Paracelsus Medical University (PMU), Bolzano, Italy; 3 Department of Radiological, Oncological and Pathological Sciences, Umberto I Hospital, Sapienza University of Rome, Rome, Italy; 4 Department of Imaging and Radiation Oncology, Fondazione Policlinico Universitario A. Gemelli IRCCS, Roma, Italy

**Keywords:** Endometrial neoplasms, Magnetic resonance imaging, Cancer staging, Lymph nodes, Multidetector computed tomography

## Abstract

**Objectives:**

To assess the reproducibility and diagnostic accuracy of the Node Reporting and Data System 1.0 (Node-RADS) for detecting pelvic nodal metastases by endometrial cancer (EC) using CT and MRI, among readers with different levels of expertise.

**Materials and methods:**

This IRB-approved, single-center retrospective study included 128 patients with EC who underwent preoperative MRI at our Institution (Jan 2020–Dec 2023). Six readers with different levels of expertise in pelvic MRI (2 dedicated pelvic radiologists, 2 residents in their fourth year of training, and 2 residents in their second year of training) independently evaluated preoperative CTs and MRIs and assigned Node-RADS scores. Inter-observer agreement and inter-method agreement were calculated. Node-RADS was compared with post-surgical pathology data.

**Results:**

At surgery, pelvic nodal metastases were detected in 12.5% of the patients. Interobserver agreement in nodal status assessment using Node-RADS varied from κ = 0.783 to κ = 0.426 using MRI, and from κ = 0.936 to κ = 0.295 using CT, with worse results among less experienced readers. MRI and CT were concordant in the N definition in 94–98% of the cases. Using MRI, the most experienced readers showed 63% sensitivity and 100% specificity in the detection of nodal metastases, compared to 44% sensitivity and 96% specificity for poorly experienced readers. Using CT, the most experienced readers showed 50% sensitivity and 100% specificity; the less experienced readers showed 43% sensitivity and 94% specificity.

**Conclusions:**

Node-RADS is a reproducible and accurate tool for locoregional nodal staging of EC, but only for readers with specific experience in pelvic imaging. MRI outperforms CT in nodal assessment.

**Key Points:**

***Question**** Preoperative assessment of nodal metastases by EC is difficult, but it may help in tailoring the best surgical approach for each patient*.

***Findings**** Node-RADS is a reliable tool for assessing the presence of pelvic nodal metastases by EC, both on CT and MRI, among experienced readers*.

***Clinical relevance**** The use of Node-RADS among experienced readers enables detection of nodal metastases with good sensitivity and excellent specificity; MRI should be preferred over CT due to its higher sensitivity*.

**Graphical Abstract:**

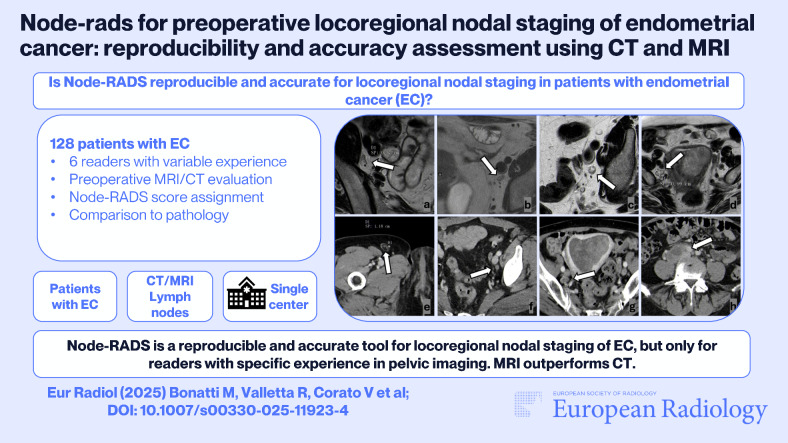

## Introduction

Endometrial cancer (EC) is the sixth most common female cancer in developed countries and the most common gynecological malignancy in Europe [1]. Its prevalence is rising due to the population’s aging and an increasing prevalence of risk factors like obesity [2]. On the other hand, because of its clinical presentation characterized by abnormal postmenopausal uterine bleeding, it is mostly diagnosed in the early stages and, consequently, mortality rates remain low, with 5-year survival rates exceeding 75% [2, 3].

EC staging is performed according to the recently revised 2023 FIGO guidelines, which introduced some new aspects, such as the subdivision of nodal metastases into micrometastases with a size of 0.2–2 mm (stages IIIC1i and IIIC2i), and macrometastases with a size larger than 2 mm (stages IIIC1ii and IIIC2ii) [4, 5]. According to the last ESGO/ESTRO/ESP guidelines, which were released before the introduction of the revised 2023 FIGO guidelines, imaging should be performed for preoperative staging of EC to tailor the best surgical approach [2]. Locoregional staging may be performed using MRI or transvaginal sonography, and both modalities ensure high accuracy in assessing the depth of myometrial infiltration and the presence of cervical stromal infiltration [6–9]. Distant staging may be performed using contrast-enhanced CT or PET-CT and is particularly useful in aggressive histological subtypes with higher metastatic potential [10, 11]. On the other hand, despite the chosen imaging modality, the diagnostic accuracy of preoperative nodal staging remains unsatisfactory, leading to a high prevalence of false-positive and false-negative results, making surgical staging crucial [12–15]. Some debate persists about the optimal surgical modality for nodal staging of EC, but currently both systematic lymphadenectomy and sentinel lymph node assessment are considered appropriate, with the second option being particularly indicated in low-risk neoplasms to reduce postoperative morbidity [2, 16–18].

**Fig. 1:**
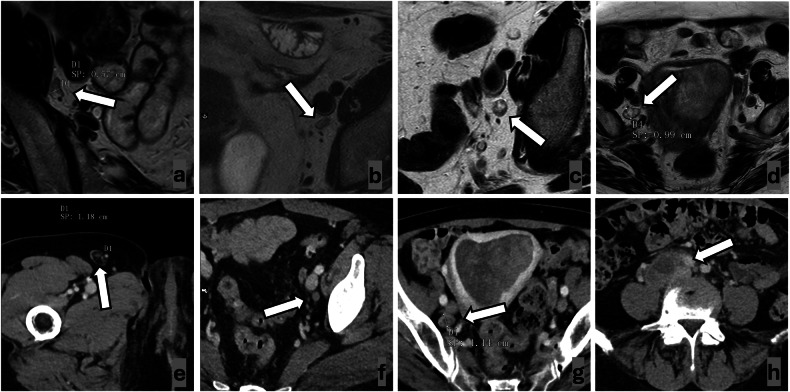
Examples of lymph nodes’ (arrows) morphological feature evaluation on high-resolution T2-weighted MR images (**a**–**d**) and on portal venous phase CT reconstructions (**e**–**h**). An ovoid shape can be observed (**a**, **b**, **d**, **e**, **g**, **h**), whereas a round shape is shown in (**c**, **f**). A preserved fatty hilum is present (**a**, **e**). Irregular margins (**b**, **f**, **g**, **h**). Heterogeneous signal intensity (**b**, **c**, **d**, **g**, **h**), with focal necrosis (**c**, **g**), and gross necrosis (**d**, **h**) (notably, **d**, **g** show the same lymph nodes, but the amount of necrosis is better appreciated on MRI than on CT)

**Fig. 2:**
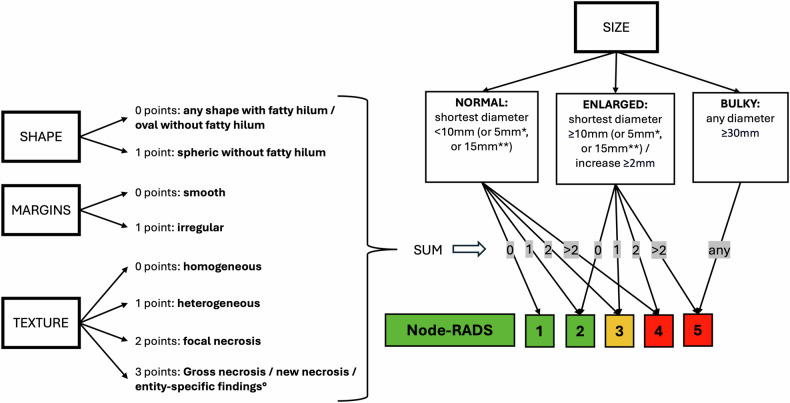
Node-RADS assessment criteria. Lymph node’s size and three different morphological criteria (shape, margins, and texture) must be evaluated. The highest score obtained for each morphological criterion must be summed; the impact of this score for Node-RADS category assignment depends on the lymph node’s size. ^*^ 5 mm in obturator, mesorectal, rectrocrural, cardio-phrenic, facial, parotid, retroauricular, occipital, retropharyngeal, and anterior jugular lymphnodes. ^**^ 15 mm in inguinal lymphnodes. ° Cystic apperaance in human papillomavirus positiv squamous cell carcinoma of the neck, thyroid cancer and non-seminomatous germ cell tumors, calcifications in thyroid cancer, and micinous texture in mucinous adenocarcinomas

To increase the accuracy and reproducibility of lymph nodes evaluation on CT and MRI, the Node Reporting and Data System 1.0 (Node-RADS) was introduced in 2021 [19]. Node-RADS is based on the combined evaluation of dimensional and morphological features (Fig. 1) and enables stratification of the risk for the evaluated lymph node to be metastatic using a 1-to-5 Likert scale (Fig. 2). In oncological staging, Node-RADS 1 and 2 are classified as N-, Node-RADS 4 and 5 are classified N+, whereas Node-RADS 3 is defined equivocal and must be interpreted according to characteristics of the primary tumor (e.g., in EC the risk of nodal metastases is very low in G1-G2 endometrioid cancers smaller than 2 cm without deep myometrial infiltration [20]). Node-RADS has been applied with variable results in many different oncological contexts, but never in preoperative staging of EC [21–27]. Moreover, inter-reader reproducibility of Node-RADS needs to be further investigated [21].

The aim of our study was to assess the reproducibility and the diagnostic accuracy of Node-RADS in preoperative staging of EC among readers with different levels of expertise, using both CT and MR images.

## Methods

### Patients’ population

**Fig. 3:**
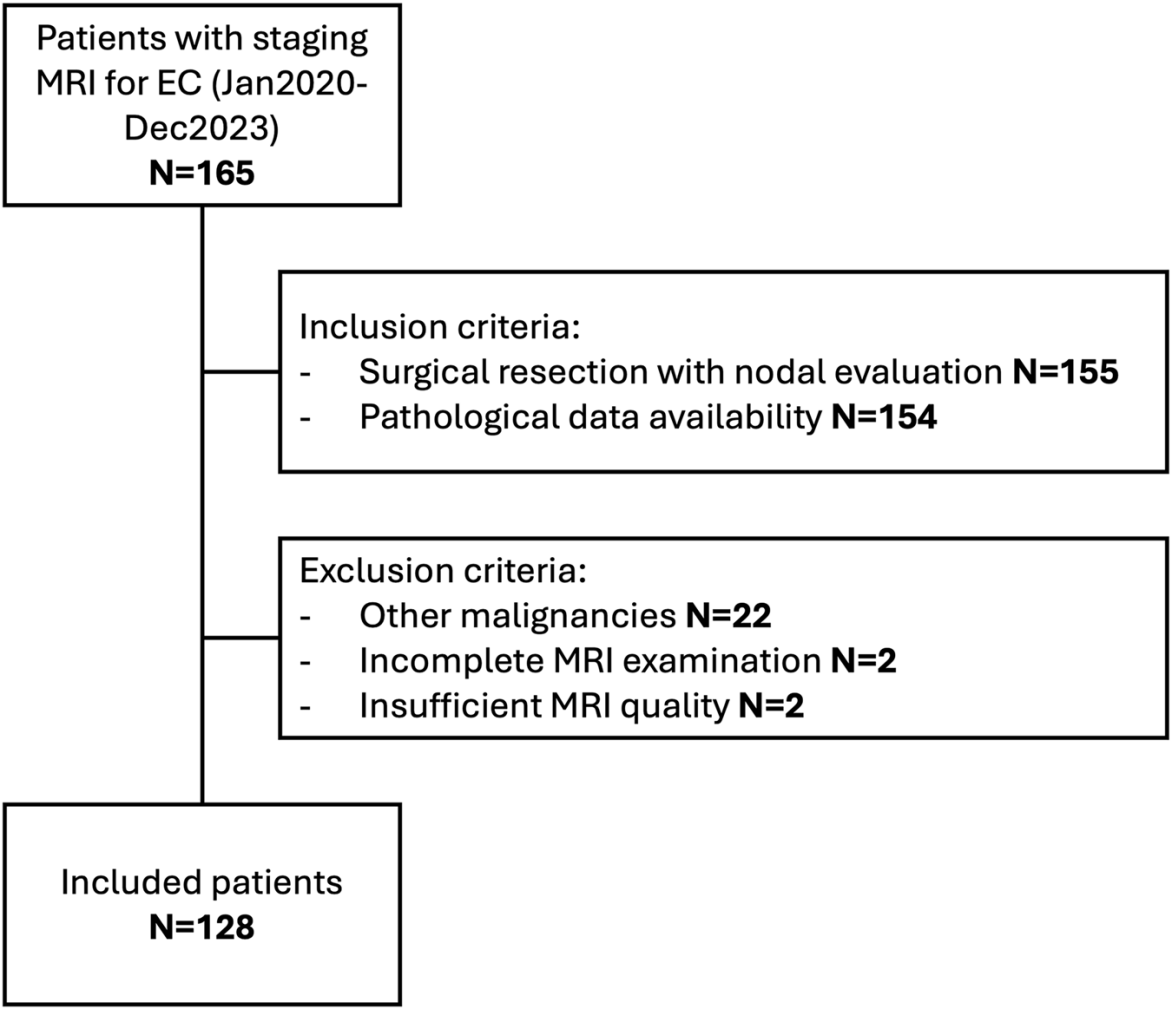
STARD flowchart for the study population

IRB-approved retrospective cohort study; the need for informed consent was waived. We considered 165 consecutive patients with histologically proven EC who underwent preoperative pelvic MRI staging at Bolzano Central Hospital between January 2020 and December 2023 for inclusion (Fig. 3). Inclusion criteria were surgical treatment at our center, including nodal staging (i.e., sentinel lymph node sampling or systematic lymphadenectomy) and the availability of post-surgical pathology data. Exclusion criteria were other coexisting or previous malignancies, incomplete MRI examinations, and insufficient MRI quality due to artifacts.

### Imaging protocols

MR examinations were performed on a 1.5-Tesla scanner (Ingenia, Philips Healthcare), using a phased array body coil; imaging parameters are reported in Table 1. Twenty mg of Hyoscine Butylbromide (Buscopan) was intravenously administered at the beginning of the scan, unless contraindicated, to reduce peristalsis-related artifacts. Intravenous contrast material (gadobutrol, 0.1 mL/kg) was administered according to the referring radiologist’s decision.

**Table 1:** MRI scanning parameters

Pulse sequence	Scanning plane	TR/TE (ms)	Voxel size (mm)	FoV (mm)
FS T2-weighted TSE	Axial (pelvis)	7700/83	1.3 × 0.9 × 5.0	400
T1-weighted TSE	Axial (pelvis)	730/10	0.9 × 0.6 × 5.0	350
T2-weighted TSE	Para-sagittal, para-axial, para-coronal (uterus)	3200/82	0.5 × 0.5 × 4.0	250
EPI (*b* = 0, 500, 1000 s/mm^2^)	Para-sagittal, para-axial (uterus)	3100/98	2.0 × 1.0 × 4.0	250
CE T1-weighted TSE	Para-sagittal, para-axial, para-coronal (uterus)	606/9.5	1.3 × 0.8 × 4.0	250

*FS* fat-saturated, *TSE* turbo spin echo, *EPI* echo planar imaging, *CE* contrast enhanced

CT examinations were performed on a dual-source 128-slice detector-row scanner (Somatom Drive, Siemens Healthineers) with patients lying supine with the arms bent over the head. Every examination included at least a portal venous phase scan of the abdomen and pelvis acquired 70 s after intravenous administration of 1.6 mL/kg of 350 mgI/mL of iodinated contrast material (Omnipaque 350, GE Healthcare); 1.5-mm-thick axial images, as well as 3-mm-thick axial, sagittal, and coronal MPR reconstructions, were sent to the PACS.

### Image analysis

Image analysis was independently performed on a workstation (Syngo.plaza, Siemens Healthineers) by six readers with different degrees of experience: two highly experienced readers (R1 and R2, radiologists with 16 years and 9 years of experience in pelvic imaging), two intermediately experienced readers (R3 and R4, residents at the end of their 4th year of training, both with 2 years of specific training in pelvic imaging), and two poorly experienced readers (R5 and R6, residents in their 2nd year of training, both without any specific experience in pelvic imaging). Intermediately and poorly experienced readers received a dedicated training session conducted by the highly experienced ones including individual and group reading of the original article by Elsholtz et al [19] and a joint review of ten CT and ten MRI examinations used for preoperative staging of EC (not included in the current study, as they were acquired prior to the inclusion period), using the Node-RADS assessment. All readers were aware of the presence of EC, as well as its histological type and grade as determined by preoperative biopsy. However, they were blinded to the final histological type and grade, as well as to any other clinical or pathological information. MRI and CT images were evaluated in different reading sessions. On MRI, DWI was used for lymph node detection, whereas T2- and T1-weighted images were used for Node-RADS score assignment. On CT, 1.5-mm-thick axial images and 3-mm-thick MPR reconstructions were used for both lymph node detection and Node-RADS score assignment. Each reader was asked to assign a Node-RADS category [19] to each MRI and CT study according to the previously described scheme (Fig. 1). Readers were instructed to assess and report lymph node size (normal/enlarged/bulky) for all cases, while the evaluation of individual morphological features was required only for lymph nodes classified as Node-RADS categories 3–5. According to the assigned scores, each exam was subdivided into N- (Node-RADS 1-2, and Node-RADS 3 in patients with G1-G2 endometrioid cancers smaller than 2 cm without deep myometrial invasion on MRI), and N+ (Node-RADS 4-5, and Node-RADS 3 in patients with G3 endometrioid cancers, or non-endometrioid cancers, or cancers larger than 2 cm, or cancers with deep myometrial invasion on MRI). Discrepancies in N staging between the two readers of each of the 3 different experience subgroups (R1–R2, R3–R4, and R5–R6) were solved by consensus.

### Statistical analysis

Continuous variables are expressed as mean ± SD or as median with interquartile range (IQR) according to their distribution. Normal distribution was assessed using the D’Agostino–Pearson test.

Kappa statistics were used to assess inter-reader concordance in Node-RADS score assessment in each experience subgroup (weighted κ), inter-reader concordance in the identification of metastatic lymph nodes in each experience subgroup (simple κ), and inter-reader concordance in the identification of metastatic lymph nodes between the different experience groups after consensus (simple κ). The chi-squared test was conducted to determine whether there was a significant difference in the number of disagreements among the reader groups. Imaging findings were compared with pathological results using the chi-square test to assess accuracy, sensitivity, and specificity.

## Results

We included in the study 128 women with a mean age of 67 ± 11 years. The patient population characteristics are reported in Table 2. One hundred out of 128 patients (78.1%) underwent both MRI and CT, whereas 28 underwent only MRI. The median interval between MRI and CT was 1 day (IQR 0–8 days). At pathology, pelvic nodal metastases were present in 16/128 (12.5%) of the cases, with macrometastases in 14/16 (87.5%) and micrometastases in 2/16 (12.5%).

**Table 2:** Patients’ population characteristics

Age (years)	67 ± 11	
Tumor type on biopsy	Endometrioid	102/128 (79.7%)
	Non-endometrioid	26/128 (20.3%)
Tumor grade on biopsy	G1	51/128 (39.8%)
	G2	37/128 (28.9%)
	G3	40/128 (31.3%)
Molecular subtype on biopsy	NSMP	56/128 (43.8%)
	MMRd	48/128 (37.5%)
	P53abn	15/128 (11.7%)
	POLE	9/128 (7.0%)
Time from MRI to surgery (days)	13 (IQR 7–20)	
Tumor maximum diameter (mm)	31 (IQR 18–45)	
Tumor type on surgical specimen	Endometrioid	104/128 (81.3%)
	Non-endometrioid	24/128 (18.7%)
Tumor grade on surgical specimen	G1	28/128 (21.9%)
	G2	53/128 (41.4%)
	G3	47/128 (36.7%)
Tumor stage (FIGO 2023)	I	64/128 (50.0%)
	II	45/128 (35.2%)
	III	16/128 (12.5%)
	IV	3/128 (2.3%)

A table reporting the Node-RADS scores assigned by each reader for each case, on both MRI and CT, is provided in Supplementary Material 1. Additional data regarding Node-RADS scoring and the subsequent assessment of nodal metastases by the six readers on MRI and CT are also available in Supplementary Materials 2 through 5. Furthermore, retrospective assessment of morphological features was performed whenever possible, i.e., for lymph nodes classified as Node-RADS 1 and for enlarged lymph nodes classified as Node-RADS 2, in which all morphological parameters were necessarily negative.

The results of the agreement analysis for Node-RADS scoring and the subsequent assessment of nodal metastases on MRI are presented in Table 3. Concordance rates for nodal size evaluation ranged from 81% to 91%, whereas concordance rates for morphological parameters evaluation, which was possible in 73–84% of the cases, ranged from 80% to 97% for nodal shape, from 92% to 96% for nodal margins, and from 80% to 93% for nodal texture. Concordance rates for the assessment of nodal metastases on MRI were 96.1% among highly experienced readers, 96.9% among intermediately experienced readers, and 89.8% among poorly experienced readers. Consensus was required in 5 out of 128 cases (3.9%) for the highly experienced group, 4 cases (3.1%) for the intermediately experienced group, and 13 cases (10.2%) for the poorly experienced group. The differences in the number of disagreements among the three groups were statistically significant (χ² = 7.0397, d*f* = 2, *p* = 0.0296). After consensus, nodal metastases were deemed present on MRI in 10 of 128 cases (7.8%) by the highly experienced readers, 9 cases (7.0%) by the intermediately experienced readers, and 11 cases (8.6%) by the poorly experienced readers.

**Table 3:** Results of the agreement analysis for Node-RADS scoring and the subsequent assessment of nodal metastases on MRI

MRI		k	95% CI
Node-RADS assignment	Highly experienced readers	0.545	0.389–0.702
	Intermediately experienced readers	0.587	0.420–0.753
	Poorly experienced readers	0.150	0.024–0.276
Nodal metastases assessment	Highly experienced readers	0.716	0.479–0.952
	Intermediately experienced readers	0.783	0.578–0.988
	Poorly experienced readers	0.426	0.170–0.682

The results of the agreement analysis for Node-RADS scoring and the subsequent assessment of nodal metastases on CT are presented in Table 4. Concordance rates for nodal size evaluation ranged from 83% to 100%, whereas concordance rates for morphological parameters evaluation, which was possible in 79–83% of the cases, ranged from 78% to 92% for nodal shape, from 88% to 95% for nodal margins, and from 72% to 88% for nodal texture. Concordance rates for the assessment of nodal metastases on CT were 96.0% among highly experienced readers, 99.0% among intermediately experienced readers, and 84.0% among poorly experienced readers. Consensus was required in 4 out of 100 cases (4.0%) for the highly experienced group, 1 case (1.0%) for the intermediately experienced group, and 16 cases (16.0%) for the poorly experienced group. The differences in the number of disagreements among the three groups were statistically significant (χ² = 19.355, d*f*  = 2, *p* < 0.001). After consensus, nodal metastases on CT were identified in 7 of 100 cases (7.0%) by the highly experienced readers, 9 cases (9.0%) by the intermediately experienced readers, and 11 cases (11.0%) by the poorly experienced readers.

**Table 4:** Results of the agreement analysis for Node-RADS scoring and the subsequent assessment of nodal metastases on CT

CT		k	95% CI
Node-RADS assignment	Highly experienced readers	0.506	0.312–0.700
	Intermediately experienced readers	0.672	0.515–0.828
	Poorly experienced readers	0.176	0.011–0.341
Nodal metastases assessment	Highly experienced readers	0.728	0.475–0.981
	Intermediately experienced readers	0.936	0.811–1.00
	Poorly experienced readers	0.295	0.037–0.554

The mean Node-RADS scores did not differ significantly between MRI and CT (*p* > 0.05). MRI and CT showed concordant assessments regarding the presence of nodal metastases in 98 out of 100 cases (98.0%) for both highly experienced and intermediately experienced readers, and in 94 out of 100 cases (94.0%) for poorly experienced readers.

When compared to pathology, MRI-defined Node-RADS achieved 62.5% sensitivity, 100% specificity, 95.3% accuracy, 100% PPV, and 94.9% NPV in the detection of nodal metastases among highly experienced readers, 50% sensitivity, 99.1% specificity, 93% accuracy, 88.9% PPV, and 93.3% NPV among intermediately experienced readers, and 43.8% sensitivity, 96.4% specificity, 89.8% accuracy, 63.6% PPV, and 92.3% NPV among poorly experienced readers. On the other hand, CT-defined Node-RADS achieved 50% sensitivity, 100% specificity, 93% accuracy, 100% PPV, and 92.5% NPV in the detection of nodal metastases among highly experienced readers, 50% sensitivity, 97.7% specificity, 91% accuracy, 77.8% PPV, and 92.3% NPV among intermediately experienced readers, and 42.9% sensitivity, 94.2% specificity, 87% accuracy, 54.5% PPV, and 91% NPV among poorly experienced readers.

## Discussion

This retrospective study aimed to evaluate the reproducibility of Node-RADS among readers with different degrees of experience, and to assess its diagnostic accuracy in detecting nodal metastases in the preoperative staging of EC on CT and MRI.

Pelvic nodal metastases were present in 12.5% of the included patients, being macrometastases in 87.5% of these cases, confirming that, in developed countries, EC is mostly diagnosed at an early stage. The prevalence of locoregional nodal metastases in our study was lower than that reported in the MAPPING study (25.6%) [13], in the ACRIN 6671/GOG 0233 Trial (20%) [28], and in the paper by Stewart et al (17%) [29], which included only high-risk cancers was in line with that reported by Fares et al in their large meta-analysis (12%) [14].

Interobserver agreement in the definition of the five different Node-RADS categories varied according to the experience of the readers and the imaging modality: highly experienced readers showed moderate agreement both on MRI and CT, intermediately experienced readers showed moderate agreement on MRI and substantial agreement on CT, whereas inexperienced readers showed slight agreement both on MRI and CT. The agreement was better when the Node-RADS categories were used to subdivide patients into N+ and N−. For this aim, highly experienced readers showed substantial agreement both on MRI and CT, intermediately experienced readers showed substantial agreement on MRI and almost perfect agreement on CT, whereas poorly experienced readers showed moderate agreement on MRI and fair agreement on CT. These results demonstrate that Node-RADS is a reproducible tool, but only among readers with specific experience in the evaluated anatomical region. Moderate to substantial agreement in Node-RADS scoring by experienced readers have already been demonstrated in many other studies, as reported in the paper by Parillo [30]. In contrast, the limitations in reproducibility among less experienced readers, although intuitively expected, have not been previously reported and warrant further investigation.

**Fig. 4:**
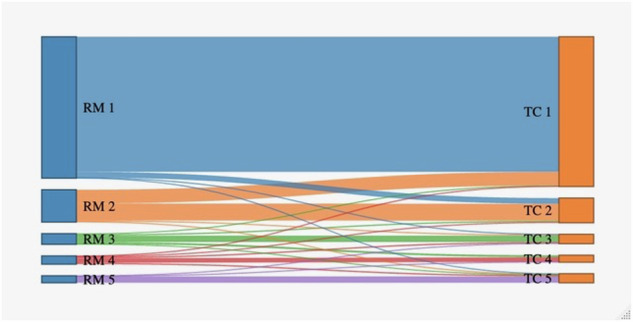
Sankey diagram showing the changes in Node-RADS score assignment between MRI and CT (all observations performed by the 6 readers are reported)

In our study Node-RADS score assignment didn’t show significant differences between MRI and CT, and the score didn’t change between the two imaging modalities in most of the cases (Fig. 4). The two imaging modalities were concordant in the assessment of nodal status in 98% of the cases both in highly and intermediately experienced readers, and in 94% of the cases in the poorly experienced readers. The best performance in detecting nodal metastases from EC using Node-RADS, independently of the imaging modality, was achieved by the highly experienced readers, who showed 95% accuracy using MRI, and 93% accuracy using CT. Intermediately experienced readers showed 93% accuracy using MRI, and 91% accuracy using CT, whereas poorly experienced readers showed 87% accuracy using MRI, and 87% accuracy using CT. Moreover, MRI showed better diagnostic performance than CT at all experience levels, with higher sensitivity and specificity. This is the first study to explore the performance of Node-RADS in locoregional nodal staging of EC. Previous studies [13, 31, 32] investigated the accuracy of morphological MRI criteria for nodal staging of EC, showing sensitivity values ranging from 46% to 70% and specificity of about 88%, as well as morphological contrast-enhanced CT criteria [28], showing sensitivity of 48% and specificity of 89%. According to our results, the application of Node-RADS criteria by experienced readers enables an increase in the negative predictive value of both CT and MRI in ruling out nodal metastases, keeping sensitivity at the highest levels. Moreover, to our best knowledge, this is the first article that directly compared Node-RADS scoring using CT and MRI head-to-head, and according to our results, MRI should be preferred due to its higher sensitivity in the detection of nodal metastases.

Our study has several limitations, primarily related to its retrospective design and to the decision to include only patients who underwent surgery. First, the number of patients with nodal metastases was relatively low, however, this reflects real-world clinical practice in developed countries. Second, only 78% of the included patients underwent both CT and MRI. Nevertheless, this subgroup included all patients with pelvic nodal metastases, and therefore, this does not affect the evaluation of diagnostic accuracy. Third, data about inter-reader agreement in nodal morphology assessment are incomplete due to the study design. Finally, some of the patients’ cohort underwent sentinel lymph node sampling instead of systematic lymphadenectomy, and consequently, we were unable to perform a “per-lymph-node” analysis. We believe this limitation is intrinsic to the methodology and currently unavoidable given the increasing use of sentinel lymph node sampling in surgical staging.

In conclusion, our study demonstrated that Node-RADS represents a reproducible and accurate tool for locoregional nodal staging of EC, but its performance is strictly dependent on readers’ experience and may be unsatisfactory for poorly experienced readers. Moreover, MRI provides higher sensitivity than CT in the detection of nodal metastases.

## Supplementary information

ELECTRONIC SUPPLEMENTARY MATERIAL

## Abbreviations

EC: Endometrial cancer
Node-RADS: Node Reporting and Data System 1.0
