# Supplementary material for: Node-RADS for preoperative locoregional nodal staging of endometrial cancer: reproducibility and accuracy assessment using CT and MRI
Source: Eur Radiol. 2025 Aug 20;36(2):989–97. doi: 10.1007/s00330-025-11923-4 (PMC12953289; doi:10.1007/s00330-025-11923-4)

Node-RADS for Preoperative Locoregional Nodal Staging of Endometrial Cancer: Reproducibility and Accuracy assessment using CT and MRI
ELECTRONIC SUPPLEMENTARY MATERIAL

Supplementary material 1. Results of Node-RADS assignment by the 6 readers on MRI and CT. Blank cells in the CT columns represent patients who didn't undergo CT.

Patient Number	R1 NODE-RADS MRI	R2 NODE-RADS MRI	R3 NODE-RADS MRI	R4 NODE-RADS MRI	R5 NODE-RADS MRI	R6 NODE-RADS MRI	R1 NODE-RADS CT	R2 NODE-RADS CT	R3 NODE-RADS CT	R4 NODE-RADS CT	R5 NODE-RADS CT	R6 NODE-RADS CT	Nodal Metastasis (0=no, 1=yes)
**1**	2	1	1	1	1	1	2	2	1	1	2	1	0
**2**	1	1	1	1	1	1							0
**3**	1	1	2	1	1	1	1	1	1	1	1	1	0
**4**	1	1	1	1	1	2	1	1	1	1	1	2	0
**5**	1	1	1	1	1	1	1	1	1	1	1	1	0
**6**	2	1	1	1	1	1	1	1	1	1	1	1	0
**7**	2	1	1	2	1	1	1	1	1	2	1	1	0
**8**	1	1	1	4	1	1							0
**9**	1	1	2	2	1	1	1	1	1	2	1	1	1
**10**	1	1	1	2	1	1							0
**11**	1	1	1	1	1	1							0
**12**	1	1	1	4	1	1	1	1	1	1	1	1	0
**13**	1	1	1	1	1	1	1	1	1	1	1	1	0
**14**	2	1	2	3	2	2	2	1	2	2	2	3	0
**15**	1	1	1	1	1	1	1	1	1	1	1	1	0
**16**	1	1	1	1	2	1	1	1	1	1	1	1	0
**17**	1	1	2	3	3	1	2	1	2	3	3	1	0
**18**	1	1	1	1	1	1	1	1	1	1	1	1	0
**19**	1	1	1	1	1	1	1	1	1	1	1	2	1
**20**	1	3	1	1	2	1	1	4	1	1	1	1	0
**21**	2	1	2	1	1	1	1	1	2	1	1	1	1
**22**	2	2	1	1	1	1	1	2	1	1	1	1	1
**23**	1	1	1	1	1	1	1	1	1	1	1	1	0

Eur Radiol (2025) Bonatti M, Valletta R, Corato V, et al.

**24**	1	1	1	1	1	2	1	1	1	1	1	2	0
**25**	5	3	4	3	4	3	3	3	3	2	4	1	1
**26**	1	1	1	1	1	1	1	1	1	1	1	1	0
**27**	1	1	1	1	1	1	1	1	1	1	1	1	0
**28**	2	3	2	2	4	1	1	3	2	1	5	1	0
**29**	1	1	1	1	1	1	1	1	1	1	1	3	0
**30**	1	1	1	1	1	2	1	1	1	1	1	2	0
**31**	1	1	1	2	2	1	1	1	1	1	1	1	0
**32**	1	1	1	1	1	4	1	1	1	1	1	3	0
**33**	1	1	1	1	1	1							0
**34**	1	3	2	1	5	1	1	3	2	1	5	1	1
**35**	1	1	1	1	1	1							0
**36**	5	3	4	3	5	3	5	4	5	3	5	5	1
**37**	5	5	5	5	5	3	5	5	5	5	5	3	1
**38**	1	1	1	1	2	1	1	1	1	1	1	2	0
**39**	1	1	1	1	2	1	1	1	1	1	1	1	0
**40**	1	1	1	1	1	1	1	1	1	1	1	1	0
**41**	1	2	3	1	1	2	2	3	3	1	1	2	0
**42**	2	2	2	1	1	3							0
**43**	1	1	1	1	1	1							0
**44**	1	1	1	1	1	1							0
**45**	5	4	5	5	3	5	5	4	5	5	4	5	1
**46**	1	1	1	1	1	1	1	1	1	1	1	1	0
**47**	2	1	1	2	4	1	2	1	1	2	4	1	0
**48**	1	1	1	1	1	1	1	1	1	1	1	1	0
**49**	1	1	2	1	1	1	1	1	2	1	1	1	0
**50**	1	1	1	1	1	1							0
**51**	2	1	2	2	4	1	1	1	2	1	4	1	0
**52**	5	3	4	4	2	5	5	3	4	4	2	5	1
**53**	1	1	1	1	1	1	1	1	1	1	1	1	0
**54**	1	1	1	1	1	1	1	1	1	1	1	1	0
**55**	1	1	1	1	1	1	1	1	1	1	2	1	0
**56**	1	2	1	1	2	1	1	2	1	1	2	2	0

**57**	2	2	2	1	3	1	2	2	2	1	3	1	0
**58**	1	1	1	1	1	1							0
**59**	1	1	1	1	1	1	1	1	2	1	1	1	0
**60**	1	1	1	1	2	1	1	1	1	1	2	1	0
**61**	1	1	1	1	1	1	1	1	1	1	1	1	0
**62**	1	1	1	1	1	2	1	1	1	1	1	2	0
**63**	1	1	1	1	2	1	1	1	1	2	2	1	0
**64**	2	2	2	2	4	2	2	2	2	2	5	1	0
**65**	1	2	1	1	4	1	1	2	1	1	4	1	0
**66**	1	1	1	1	1	1							0
**67**	1	1	1	1	1	2	1	1	1	1	1	2	0
**68**	1	1	1	1	1	1							0
**69**	1	3	2	2	1	1							0
**70**	1	1	1	1	1	1	1	1	1	1	1	1	0
**71**	1	1	1	1	1	1							0
**72**	1	1	1	1	1	1							0
**73**	1	1	1	1	1	1	1	1	2	1	1	1	0
**74**	1	1	1	1	1	1	1	1	1	1	2	1	0
**75**	1	1	1	1	1	2							0
**76**	1	1	1	1	1	2	1	1	1	1	1	1	0
**77**	1	1	1	2	1	1	1	1	1	1	1	1	0
**78**	1	1	1	1	1	3							0
**79**	1	1	2	1	1	2	1	1	5	5	1	5	0
**80**	1	2	2	3	2	2							0
**81**	1	1	1	1	1	1							0
**82**	1	1	1	1	1	1							0
**83**	2	1	1	1	1	2	1	1	1	1	1	1	0
**84**	1	1	1	1	1	3	1	1	1	1	1	3	0
**85**	1	2	1	1	1	3							0
**86**	1	1	1	1	2	1	1	1	1	1	1	1	0
**87**	1	1	1	1	1	2	1	1	1	1	1	1	0
**88**	1	1	1	1	1	1							0
**89**	1	1	1	1	1	1	1	1	1	1	1	2	0

**90**	1	1	1	1	1	1	1	1	1	1	1	1	0
**91**	1	2	2	1	2	4	1	2	2	1	1	3	0
**92**	1	1	2	1	1	2	1	1	2	1	1	1	0
**93**	2	3	1	4	2	4	1	3	1	2	2	4	0
**94**	1	1	1	1	3	2	1	1	1	1	2	2	0
**95**	2	2	2	2	3	3	1	2	2	2	3	4	0
**96**	1	1	2	1	1	2	1	1	2	1	1	1	0
**97**	5	1	5	5	4	1	5	1	5	5	4	2	1
**98**	2	4	2	2	4	3	1	5	3	2	4	3	0
**99**	1	1	1	1	1	1	1	1	1	1	1	1	0
**100**	1	1	1	1	1	1							0
**101**	1	1	1	1	1	1	1	1	1	1	1	1	0
**102**	1	1	1	1	1	1	1	1	1	1	1	1	1
**103**	1	1	1	1	1	1	1		1	1	1	1	0
**104**	1	1	1	1	1	2	1	1	1	1	1	1	0
**105**	1	1	1	1	1	1							0
**106**	5	5	3	4	5	3	4	5	3	3	5	3	1
**107**	1	1	1	1	3	1	1	1	1	1	3	1	0
**108**	2	2	3	1	2	3	1	1	2	2	1	1	0
**109**	4	1	3	1	3	1	3	1	2	1	2	3	1
**110**	1	1	1	1	1	1	1	1	1	1	1	1	0
**111**	1	1	1	1	2	1							0
**112**	1	1	1	1	1	1							0
**113**	1	1	1	1	2	1							0
**114**	4	4	4	4	3	1	4	4	4	4	4	1	1
**115**	1	2	2	1	1	2	1	2	2	1	1	2	0
**116**	1	1	1	1	3	1	1	1	1	1	3	1	0
**117**	1	1	1	1	1	2	1	1	1	1	1	2	1
**118**	1	1	1	1	1	1	1	1	1	1	1	1	0
**119**	2	2	1	1	3	2	1	2	1	1	4	2	0
**120**	1	1	1	1	1	1	1	1	1	1	1	1	0
**121**	1	1	1	1	1	1	1	1	1	1	1	1	0
**122**	1	1	1	1	1	2	1	1	1	1	1	1	0

**123**	2	2	1	1	1	1	1	2	1	1	1	1	0
**124**	1	1	1	1	1	1	1	1	1	1	1	1	0
**125**	1	1	1	1	1	2	1	1	1	1	1	1	0
**126**	1	1	1	1	1	1	1	1	1	1	1	2	0
**127**	2	3	2	2	2	2	1	3	1	1	1	2	0
**128**	1	1	1	1	1	1	1	1	1	1	1	1	0

Supp 2. Results of Node-RADS assignment by the 6 readers on MRI.

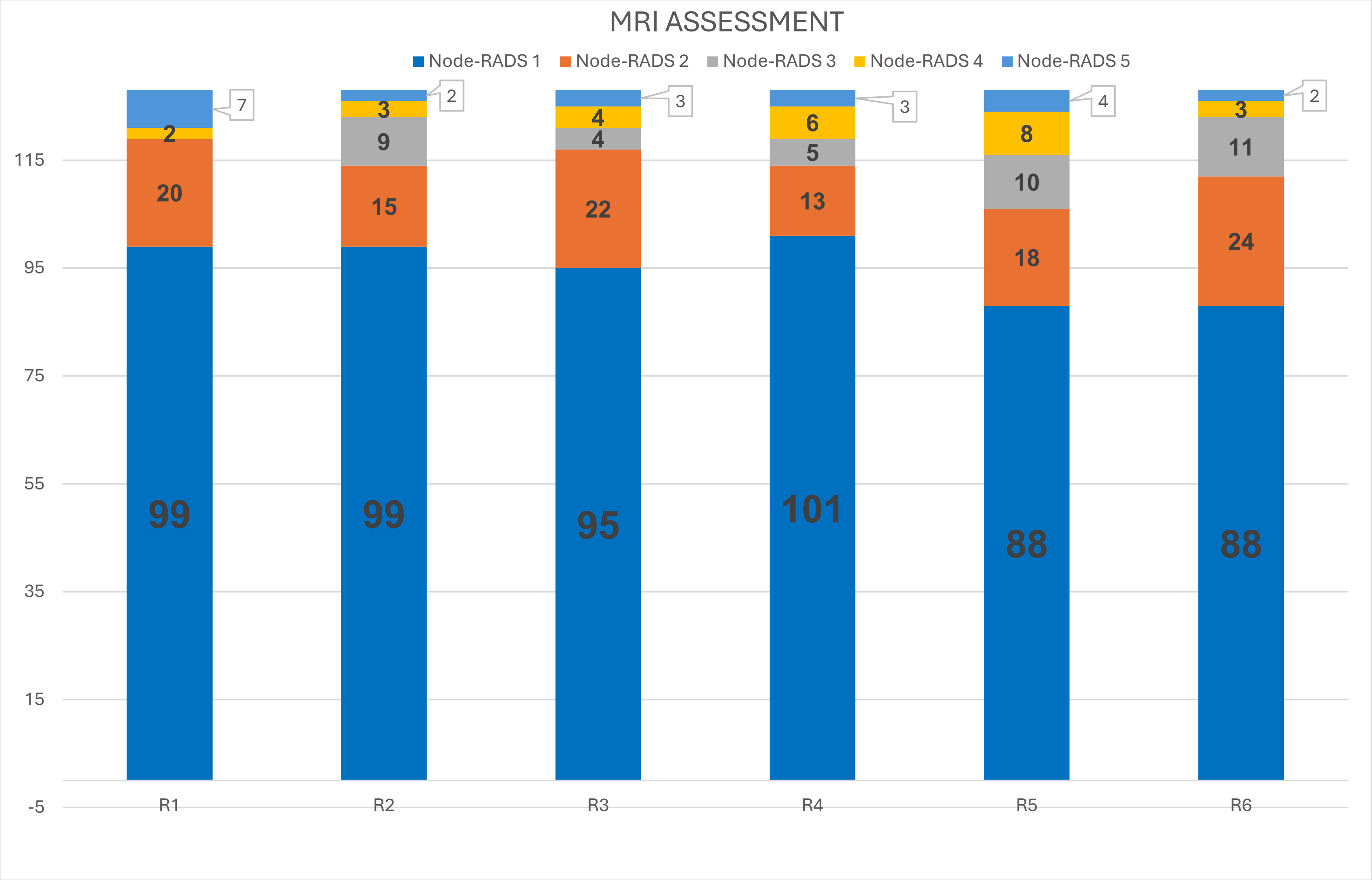

Eur Radiol (2025) Bonatti M, Valletta R, Corato V, et al.

Supp 3. Results of nodal metastases assessment by the 6 readers on MRI.

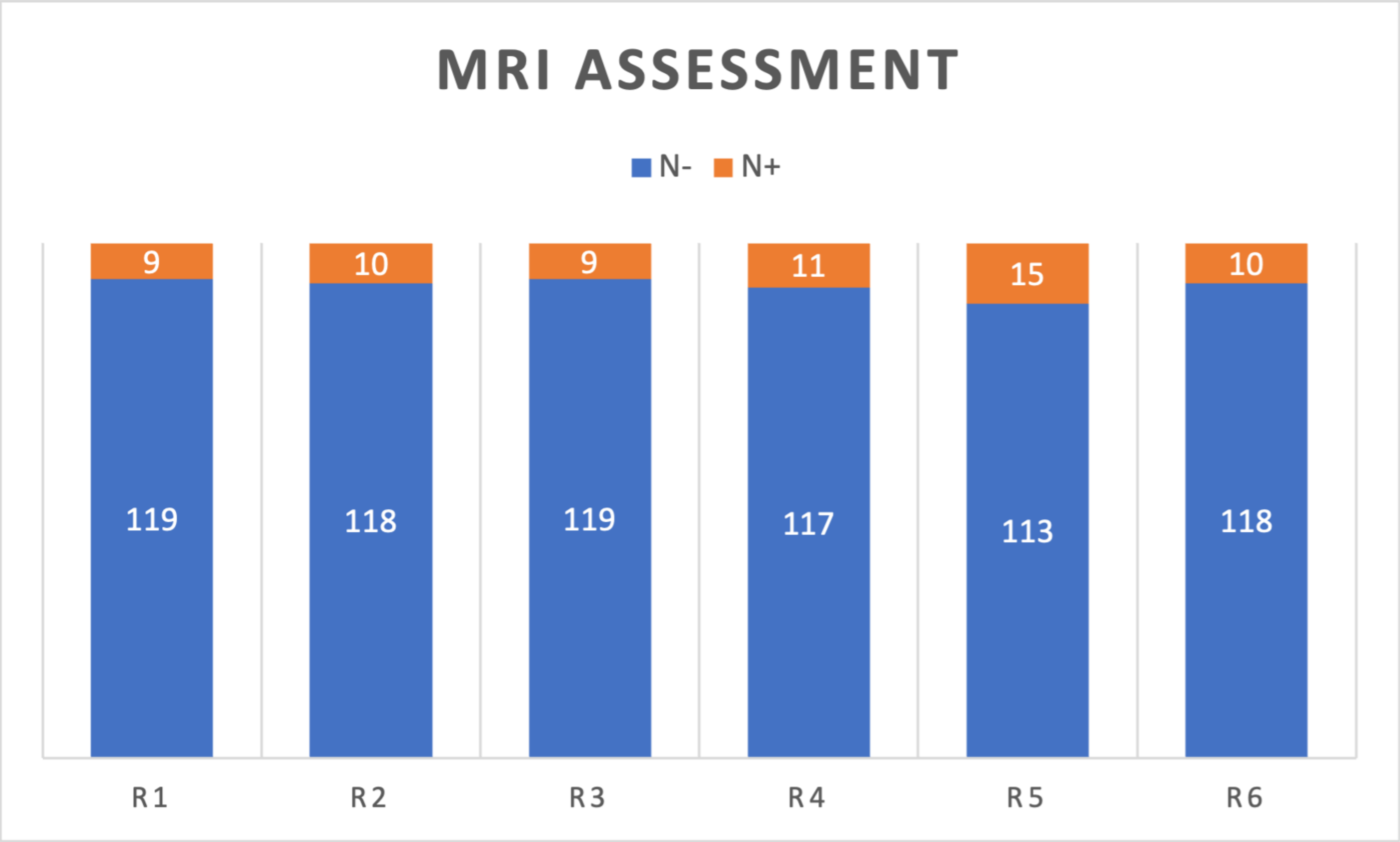

Supp 4. Results of Node-RADS assignment by the 6 readers on CT.

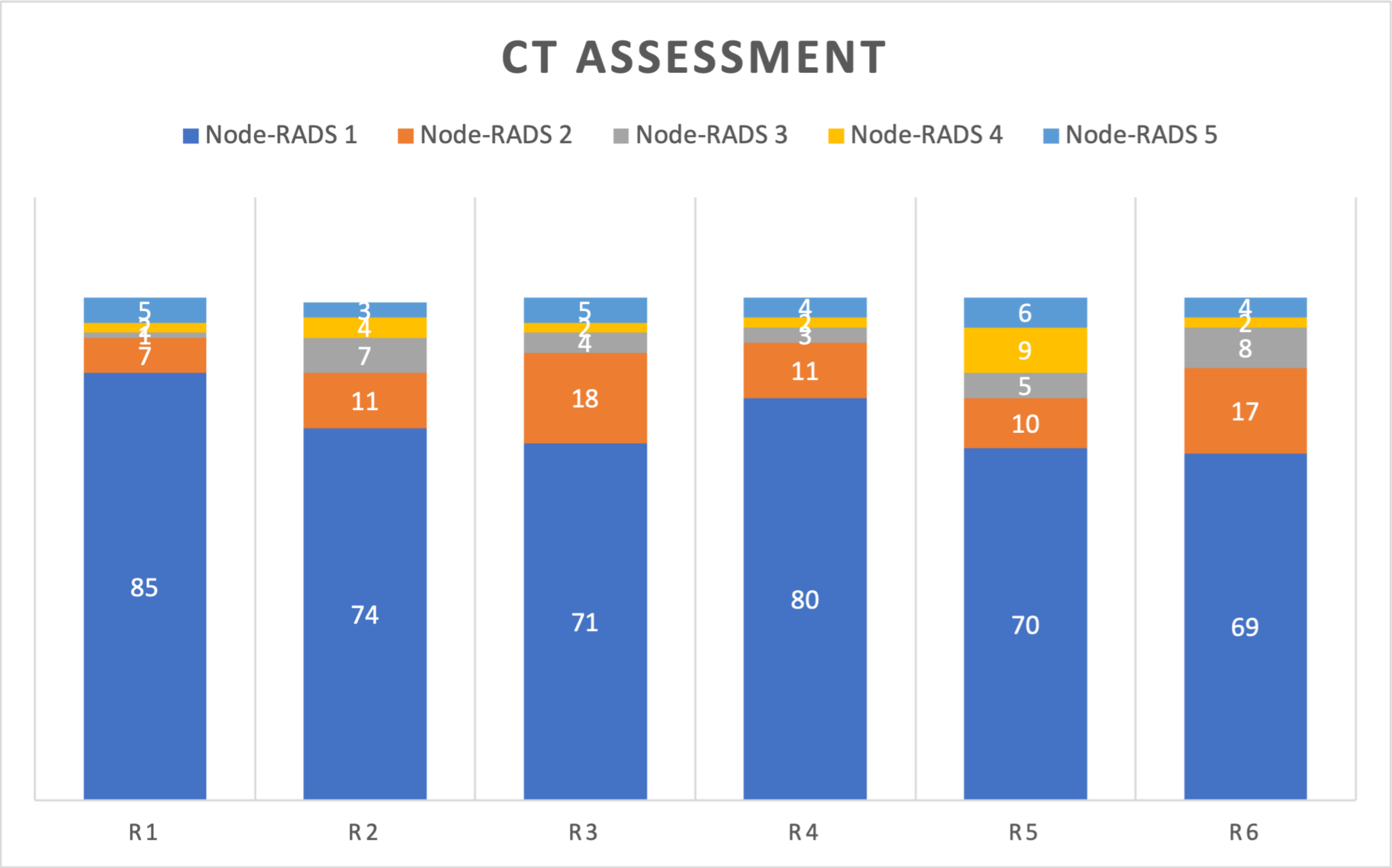

Eur Radiol (2025) Bonatti M, Valletta R, Corato V, et al.

Supp 5. Results of nodal metastases assessment by the 6 readers on CT.

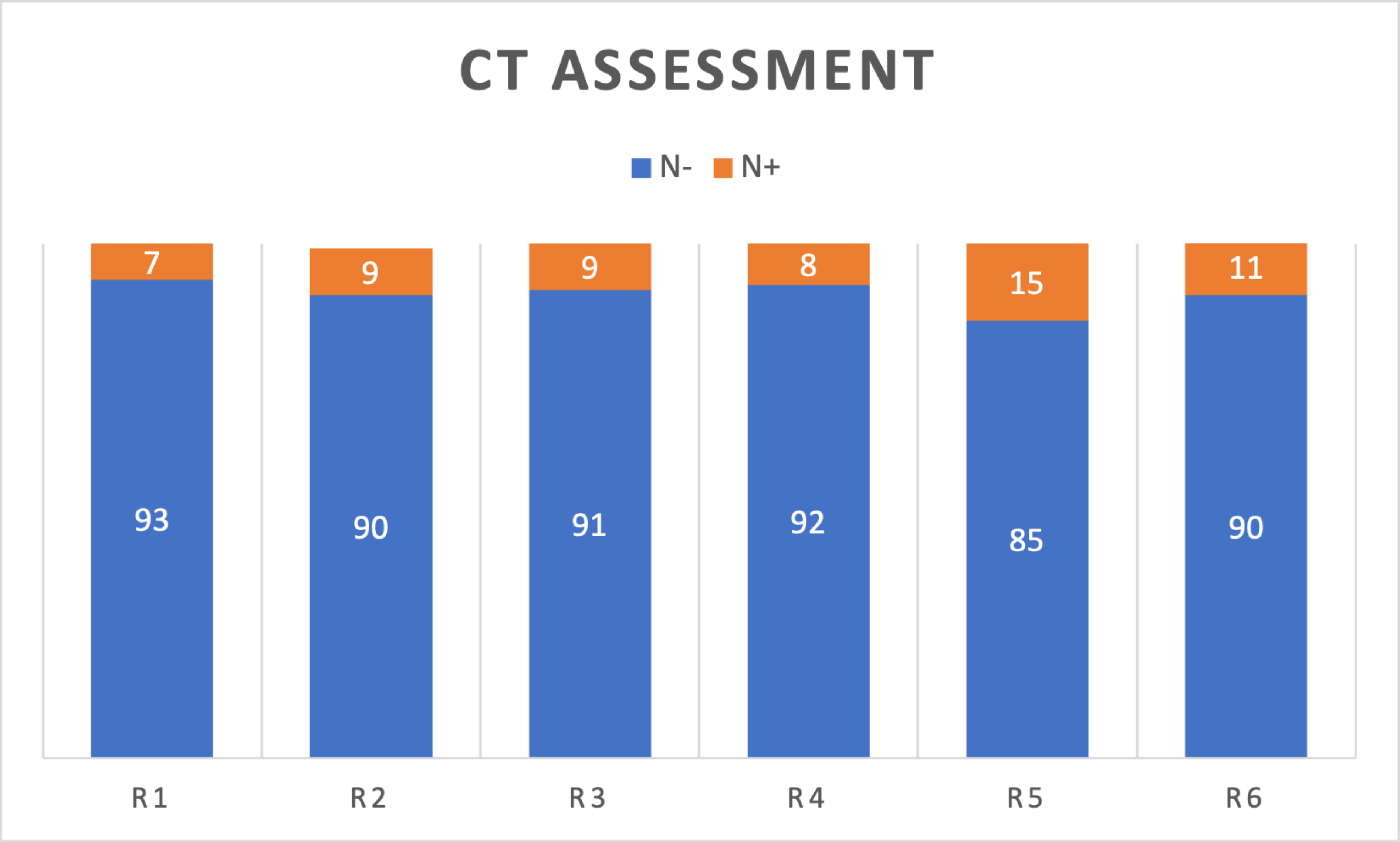

Supplement: Supplementary file 1 — ELECTRONIC SUPPLEMENTARY MATERIAL [file 330_2025_11923_MOESM1_ESM.pdf]
